# Acculturative family distancing: Impact on Latinx/é grandchildren’s caregiving tendencies toward grandparents

**DOI:** 10.1093/geroni/igaf122.3292

**Published:** 2025-12-31

**Authors:** Elisa Gomez, Mayra Bámaca

**Affiliations:** University of California, Merced, Merced, California, United States; University of California, Merced, Merced, California, United States

## Abstract

Affectual solidarity is a vital indicator of relationship quality and caregiving practices from the grandchild towards the grandparent (Mansson, 2022). Caregiving practices toward grandparents capture the activities grandchildren participate in with their grandparents such as helping with chores and going to doctor’s appointments (Roberto & Stroes, 1992). Given the importance placed on family interconnectedness among Latinx/é families, grandchildren as caregivers are promising. However, the cultural distance between grandchildren and grandparents (i.e., acculturative family distance [AFD]) may play a role in caregiving tendencies. Existing studies have not explored grandchildren as caregivers nor what predicts a grandchild’s caregiving tendencies and none, to our knowledge, are dedicated to the relationship between Latinx/é grandchildren and grandparents. Using regression analysis, we investigated 1) whether perceived affection from grandparents and AFD, defined as communication incongruence, predicted participation with grandparents and 2) whether AFD was a moderator. We collected survey data from 399 young adults (Mage = 20.28; 18% males) in the California Central Valley region. Findings indicated that perceived affection from their grandparent predicted greater grandchild participation with their grandparent (β=.358). However, this association was weaker for grandchildren with higher levels of AFD (β=-0.086). AFD can lead to interrupted cultural transmission and retention in the next generation, and less social interaction and intimacy between grandparents and grandchildren (Hwang, 2006; Silverstein & Chen, 1999). Thus, AFD between generations can result in less participation with grandparents and potentially less obligation toward caregiving practices. Future research should explore how AFD can impact grandchildren’s role as potential family caregivers.

